# Amygdala fMRI Signal as a Predictor of Reaction Time

**DOI:** 10.3389/fnhum.2016.00516

**Published:** 2016-10-13

**Authors:** Philipp Riedel, Mark J. Jacob, Dirk K. Müller, Nora C. Vetter, Michael N. Smolka, Michael Marxen

**Affiliations:** Section of Systems Neuroscience, Department of Psychiatry and Neuroimaging Center, Technische Universität DresdenDresden, Germany

**Keywords:** reaction time, threat conditioning, trait anxiety, amygdala, fMRI

## Abstract

Reaction times (RTs) are a valuable measure for assessing cognitive processes. However, RTs are susceptible to confounds and therefore variable. Exposure to threat, for example, speeds up or slows down responses. Distinct task types to some extent account for differential effects of threat on RTs. But also do inter-individual differences like trait anxiety. In this functional magnetic resonance imaging (fMRI) study, we investigated whether activation within the amygdala, a brain region closely linked to the processing of threat, may also function as a predictor of RTs, similar to trait anxiety scores. After threat conditioning by means of aversive electric shocks, 45 participants performed a choice RT task during alternating 30 s blocks in the presence of the threat conditioned stimulus [CS+] or of the safe control stimulus [CS-]. Trait anxiety was assessed with the State-Trait Anxiety Inventory and participants were median split into a high- and a low-anxiety subgroup. We tested three hypotheses: (1) RTs will be faster during the exposure to threat compared to the safe condition in individuals with high trait anxiety. (2) The amygdala fMRI signal will be higher in the threat condition compared to the safe condition. (3) Amygdala fMRI signal prior to a RT trial will be correlated with the corresponding RT. We found that, the high-anxious subgroup showed faster responses in the threat condition compared to the safe condition, while the low-anxious subgroup showed no significant difference in RTs in the threat condition compared to the safe condition. Though the fMRI analysis did not reveal an effect of condition on amygdala activity, we found a trial-by-trial correlation between blood-oxygen-level-dependent signal within the right amygdala prior to the CRT task and the subsequent RT. Taken together, the results of this study showed that exposure to threat modulates task performance. This modulation is influenced by personality trait. Additionally and most importantly, activation in the amygdala predicts behavior in a simple task that is performed during the exposure to threat. This finding is in line with “attentional capture by threat”—a model that includes the amygdala as a key brain region for the process that causes the response slowing.

## Introduction

There is great variability in reaction times (RTs) between and within participants, even in RT tasks where a simple motoric reaction to a cue is required (SRT task) or in RT tasks with two possible stimuli and two possible simple reactions (CRT task) (Rabbitt et al., 2001; Salthouse and Berish, 2005). Differences in age (Der and Deary, 2006), sex (Landauer et al., 1980), practice (Ando et al., 2004), wakefulness (Van Den Berg and Neely, 2006), respiratory cycle (Buchsbaum and Callaway, 1965), personality type (Welford, 1980; Stelmack et al., 1993), and contextual factors (Bishop et al., 2009) such as threat (Johanson, 1922) likely contribute to this variance.

Threat as a signal relevant for survival (Ohman et al., 2001) typically captures attention. This capture of attention can cause a disruption in the ongoing task and can, therefore, influence RTs. Experiments that addressed the influence of threat on RT tasks yield divergent results. The continuous threat of an electric shock has been shown (i) to either enhance task performance (Weiss, 1965; Eason et al., 1969; McDonald et al., 2015) or (ii) not to affect RTs (Farber and Spence, 1956; Choi et al., 2012). However, spontaneous arousal has been demonstrated to result in slower RTs (Nishisato, 1966). When threat-conditioned stimuli are presented as transient distractors in RT tasks, faster RTs (Notebaert et al., 2011), slower RTs (Schmidt et al., 2015) or no significant difference in RTs (Koster et al., 2005a) have been observed when compared to unconditioned distractors. This variance can be partially, but not entirely explained by the influence of experimental phase (i.e., acquisition or extinction; Koster et al., 2005a). Even greater divergence, that is, slower, faster, and no significant difference in RTs in response to threat, has been observed when presenting threatening pictures as distractors (Kirita and Endo, 1995; Northoff et al., 2000; Fox et al., 2001; Buodo et al., 2002; Leppanen et al., 2003; Bishop et al., 2004; Blair et al., 2007; Soares et al., 2009). In these cases, external factors like (i) awareness of threat (Mogg and Bradley, 1999) and (ii) whether threat affects oneself or a third party (Fernandes et al., 2013) contribute to variability. In addition to SRT and CRT tasks, visual search paradigms (Ohman et al., 2001; Schmidt et al., 2015), Stroop tasks (Macleod, 1991; Mama et al., 2013), dot-probe tasks (Frewen et al., 2008; Kappenman et al., 2015), and further designs (for a review, see Gyurak et al., 2011) have been used to assess the effect of threat on ongoing cognitive processing. Methodological differences may explain the observed differences in those tasks to some extent. For example, explicit tasks where participants are highly aware of the emotional content of stimuli (e.g., a picture of a spider) and the triggered emotion (e.g., fear) may show a larger variability of RTs than implicit tasks where awareness of the emotional content and therefore potential cognitive regulation is low (for a review, see Gyurak et al., 2011). Additionally, variations within one task type (e.g., visual search paradigms) that utilize engagement effects (e.g., a threatful stimulus within neutral stimuli captures attention and is faster to find; “pop-out” effect) or effects of decelerated disengagement (e.g., threatful stimuli surrounding a neutral stimulus hold attention and increase RTs to the latter) result in different RTs (Rinck et al., 2005). Moreover, the trial-to-trial structure influences RTs in more complex tasks (e.g., congruency effects in the Stroop task, i.e., faster RTs to incongruent trials after incongruent trials compared to after congruent trials; Bugg et al., 2008). Furthermore, type of RT response (e.g., button-press or eye-movement-detection; Rinck et al., 2005), types of stimuli (e.g., pictures that are processed quickly or words that require higher cognitive processing), differences in the emotional salience of stimuli (Mama et al., 2013) and sample characteristics (e.g., high anxious participants, low anxious participants, patient with an anxiety disorder) could yield variable RTs in tasks assessing the effect of threat on behavioral performance.

In the attempt to further explain variability in RTs during the exposure to threat, previous research identified anxious personality as a predictor. Already Kamin and Clark (1957) reported that the higher the trait anxiety (Taylor Scale), the slower were RTs both for a SRT task without threat and a task motivated by avoidance of shock. However, another study did not find an effect of trait anxiety (Taylor Scale) on RT tasks (Farber and Spence, 1956). Later, differences in (i) awareness and (ii) sensitivity of the experimental design were identified to explain inconsistent effects of trait anxiety on RTs. Etkin et al. (2004) reported that participants with high trait anxiety responded faster toward fearful compared to neutral face distractors. This effect was only true for masked faces as distractors, that is, in case of unaware or indirect threat. Koster et al. (2005b) showed that differences in attentive processing of threat in high-anxious and low-anxious individuals (State-Trait Anxiety Inventory, STAI-T score) are subtle. But they were able to detect those subtle differences by (i) using pictures that varied in negative valence and arousal value and by (ii) presenting pictures at different durations. Hence, trait anxiety explains variability of RTs in threat-related conditions.

Trait anxiety has been associated with amygdala volume, its neuronal activity, its connectivity, and its perfusion (Etkin et al., 2004; Baur et al., 2012; Eden et al., 2015; Greening and Mitchell, 2015; Kaczkurkin et al., 2016) as well as activity alterations in other brain regions linked to threat processing, for example, the hippocampus (Satpute et al., 2012) and the bed nucleus of the stria terminalis (BNST; Somerville et al., 2010). Also processing of threat in the brain irrespective of personality trait has been causally linked to the amygdala (Labar et al., 1995; Bach et al., 2015). Functional magnetic resonance imaging (fMRI) research has consistently demonstrated that blood-oxygen-level-dependent (BOLD) response in the amygdala is increased in threat compared to neutral conditions (Phelps et al., 2001; Vuilleumier et al., 2001; reviewed in LeDoux, 2003) and so have other methodological approaches (Oya et al., 2002; Pissiota et al., 2003; Dumas et al., 2013; Murray et al., 2014; Stujenske et al., 2014). In addition to the amygdala, an extended network including, for example, the BNST, the hippocampus, the ventromedial prefrontal cortex (vmPFC), the insula and the primary motor cortex, has been identified for the processing of sustained threat (Walker et al., 2003; Sullivan et al., 2004; Kalisch et al., 2006; Alvarez et al., 2011; Lonsdorf et al., 2014; Andreatta et al., 2015; Herrmann et al., 2016).

Hence, we conclude, that neuronal activity, similarly to trait anxiety scores, may also predict RTs. BOLD signal *per se* has already been introduced as predictor of task performance (Musso et al., 2006). In this regard, we specifically propose neuronal activity within the amygdala to predict RTs. This conclusion has been partially confirmed by van Reekum et al. (2007). In their study, mean activity within the amygdala was increased in trials showing negative pictures compared to trials showing neutral pictures. Additionally, they showed that larger differences in mean amygdala activity between conditions are related to faster mean RTs for negative compared to neutral pictures. However, neither trial-by-trial RT-BOLD correlations to phasic threat nor RT-BOLD correlations during the exposure to sustained threat have been reported for the amygdala to our knowledge. Note, that we do not exclude that other specific brain regions or a network of different brain regions potentially function as a predictor of RTs as well (see Discussion).

**FIGURE 1:**
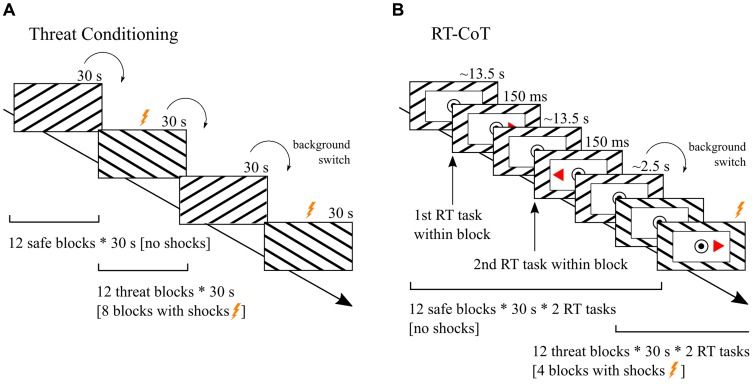
**Experimental design.** **(A)** Before the reaction time (RT) experiment [RT under Continuous Threat task (RT-CoT task)] all participants underwent *Threat Conditioning*. They were instructed that they would be receiving electric shocks while looking at a computer screen. Subsequently they were passively exposed to 24 blocks of alternating background images lasting 30 s each. Images were diagonally striped in different directions (12 with ascending and 12 with descending stripes). Only during the presentation of one of the two images ([CS+]; threat condition) participants received aversive electric shocks ([US], 8 out of 12 threat blocks). During the presentation of the other image no electric shocks were delivered ([CS-]; safe condition). Participants were not informed that electric shocks would only be applied during one of the background images. **(B)** During the RT-CoT task, again, 24 blocks of the same background images (12 indicating threat [CS+] and 12 indicating safety [CS-]) lasting 30 s each were presented alternately. In contrast to threat conditioning a white rectangle was placed in the middle of the background image to present a choice reaction time task. In the RT-CoT task, participants were instructed to indicate as fast as possible by button press in which direction a red triangle pointed. Shocks were administered in 4 out of 12 threat blocks to prevent fear extinction.

In the current study, participants first underwent threat-conditioning (**Figure 1A**). One neutral stimulus (background image, conditioned stimulus [CS+]) was paired with an aversive event [i.e., electric shock to the skin, unconditioned stimulus (US)]. During the presentation of a second neutral background image no shocks were delivered [CS-] (for a review, also see LeDoux, 2014). Afterward, participants performed a CRT task in the MRI scanner. During the task participants were—in alternating 30 s blocks—exposed to the background indicating potential shock (threat condition; [CS+]) and to the background indicating no chance of shock (safe condition; [CS-]) (**Figure 1B**). According to the presented literature (Phelps et al., 2001; Koster et al., 2005b; Musso et al., 2006; van Reekum et al., 2007; McDonald et al., 2015), we addressed three hypotheses:

(1) RTs in a CRT task will be faster in the threat condition compared to the safe condition in individuals with higher trait anxiety (as assessed with the STAI).
(2) The BOLD signal from the amygdala (indicative of neural activity) will be higher in the threat condition compared to the safe condition (i.e., control condition).
(3) BOLD activity in the amygdala prior to a CRT trial will be correlated with the corresponding RT.

## Materials and Methods

The study was approved by the Ethics Committee at the Technische Universität Dresden, Germany (EK 56022012) and carried out at the Neuroimaging Center of the Technische Universität Dresden.

### Participants

Fifty participants were recruited for the study. They all showed proficiency in the German language (telephone interview), were right-handed (as assessed with the Edinburgh Handedness Inventory; Oldfield, 1971) and reported normal or corrected-to-normal vision. They were screened for exclusion criteria for MRI. Participants had no history of mental disorder or neurologic disease as assessed with a customized, structured interview based on Diagnostic and Statistical Manual of Mental Disorders (DSM) IV criteria. Participants reported no pregnancy. Written informed consent was obtained from each participant. All were unaware of the hypotheses of the study and received payment for their participation. Five participants had to be excluded prior to analysis, because of either (i) having difficulties understanding the instructions, (ii) falsely using the wrong combination of buttons on the response box, (iii) malfunction of behavioral data recording, or (iv) technical difficulties during the conditioning phase. Forty-five participants were included in the statistical analysis of the behavioral data (19–39 years of age, *M* = 24.4, SD = 4.1, 23 female). Because of deficient measurement of peripheral physiological parameters in nine participants, less participants (*N* = 36, *M* = 24.5, *SD* = 4.2, 18 female) were included in the analysis of the neuroimaging data, which was performed after correction for physiological noise (see Materials and Methods).

### Experimental Design

#### Overview

The study reported here was part of a broader research project and was performed on the 1st day of a 5-day experimental design (Marxen et al., 2016). We will refer to this particular experimental task as RT under Continuous Threat task (RT-CoT task). Previous to the RT-CoT task, participants performed another RT task that measured attentional capture effects by distractor images of varying emotional valence. Subsequently, the following procedural steps were conducted inside the MRI scanner. First, the participants’ individual pain threshold for an electric shock of the skin was measured. Then, in the threat conditioning phase (**Figure 1A**), electrical stimulation (US) was paired with a particular background image (LaBar et al., 1998; Rhudy and Meagher, 2000). Next, participants performed a CRT task (**Figure 1B**). During the task participants were—in alternating 30 s blocks—exposed to the background indicating potential shocks (threat condition; [CS+]) and to the background indicating no chance of shock (safe condition [CS-]). Finally and outside the MRI environment, threat conditioning and personality trait were evaluated by a set of questionnaires. Visual Stimuli were presented via a digital light processing (DLP) projector (Acer Group Inc., Taipei, Taiwan) and a back-projection screen and were visible to the participant via a mirror mounted on the head coil. Stimuli were presented and responses were recorded using the software Presentation Version 16.3 (NeuroBehavioral Systems, Albany, CA, USA).

#### Electrical Stimulation and Determining Pain Threshold

For the application of electric shocks (rectangular pulses of 1 ms duration with a frequency of 100 Hz for a period of 1 s) a DS5 Isolated Bipolar Constant Current Stimulator (Digitimer Ltd, Welwyn Garden City, UK) and adhesive MRI-compatible electrocardiography (ECG) electrodes (Ambu GmbH, Bad Nauheim, Germany) were used. First, electrodes were placed on the back of the participants’ left hand. Participants were reminded about receiving electric shocks and instructed to report the perceived intensity (felt nothing—not unpleasant—unpleasant—very unpleasant) at each time via a four-button response box (Current Designs Inc., Philadelphia, PA, USA). Stimulation intensity was carefully increased in a logarithmic fashion until the participant indicated a pain intensity of at least “unpleasant” two times in a row. The corresponding stimulation intensity (1–20 mA) marked the stimulation threshold.

#### Threat Conditioning

Before the threat conditioning phase, participants were instructed that they would be receiving electric shocks while attending to a computer screen. Subsequently, participants were passively exposed to 24 blocks of alternating background images (**Figure 1A**). Images were diagonally black and white striped, with ascending or descending stripes and a width ratio of 1:10 (**Figure 1A**). Only during the presentation of one of the two images ([CS+]; threat condition) participants received an aversive stimulus ([US], i.e., pain induced by an electric shock). During the presentation of the other image participants received no shocks ([CS-]; safe condition). Image attribution to condition was pseudo-randomized with 19 participants receiving the ascending version and 26 participants receiving the descending version (**Figure 1A**) as CS+. Electric shocks were administered in 8 out of 12 threat blocks. The first threat block was always paired with a shock. In two of the eight blocks, two shocks were administered, while only one shock was administered in the other six at pseudo-random times. This resulted in a total of 10 shocks. Participants were not told that electric shocks would only be applied during one of the background images (for the difference of conditioned fear vs. instructed fear, also see Alvarez et al., 2011).

#### RT-CoT Task

In this phase, participants performed a CRT task. During the task participants were—in alternating 30 s blocks (threat condition/safe condition)—exposed to the same background images used during threat conditioning. The safe condition was always presented first. In contrast to the threat conditioning phase a white rectangle was placed on top of the center of the background image to present the CRT task. Importantly, the background image was still clearly visible (**Figure 1B**). During the RT-CoT, shocks were administered in 4 out of 12 threat blocks at pseudo-random times (i.e., at ∼3 s, ∼7 s, ∼11 s, ∼5 s, and ∼9 s prior to CRT task onset). In one of the threat blocks two shocks were administered, in the other three only one shock was administered. This resulted in a total of five shocks.

Two RT trials were included per block (24 trials per condition in total). Task onsets were at ∼13.2 s and at ∼26.4 s after the block onset. A red triangle pointing to the left or right would appear on the screen for 150 ms. Participants were asked to indicate as fast as possible in which direction the triangle pointed by pressing a button with the right index finger for “left” or with the right middle finger for “right” (**Figure 1B**). Responses were recorded via two buttons of a four-button response box (Current Designs Inc., Philadelphia, PA, USA). Participants did not receive feedback concerning RTs and correct/incorrect responses.

#### Questionnaires

After the RT-CoT task, participants completed the STAI (Kendall et al., 1976), Perceived Stress Scale (PSS; Cohen et al., 1983), and Beck Depression Inventory (BDI II; Beck et al., 1996). Including the PSS and BDI in addition to the STAI served to address the consistency of the participants’ answers; the PSS and BDI are found to correlate with the STAI (Endler et al., 1992; Lee, 2012; Isobe et al., 2014).

#### Evaluation of Threat Conditioning

After the end of the experiment, participants were asked to indicate on a 4-point Likert scale in an anxiety rating how anxious they have been (not anxious at all—a little anxious—quite anxious—very anxious) during the presence of one background (verbally described as background with ascending stripes) and during the presence of the other background (verbally described as background with descending stripes). Skin conductance was not recorded in the current study, because skin conductance measures failed to indicate an effect of condition or general anxiety in a pilot study.

### Data Analysis—Behavioral

To test whether RTs are faster in the threat condition compared to the safe condition (Hypothesis 1), we analyzed the acquired RT data. The five RT trials that followed the administration of a shock (i.e., at ∼3 s, ∼7 s, ∼11 s, ∼5 s and ∼9 s after shock onset) were not included. RTs shorter than 200 ms and longer than three standard deviations from individual means (Whelan, 2010) and incorrect responses were excluded, that is, treated as misses. The first RT trial was excluded in all participants (see Supplementary Material for a detailed rationale). Mean and median RTs were analyzed (Whelan, 2010). No differentiation of misses (i.e., in excluded vs. in actual misses) was made, because misses were rare (see Results).

Koster et al. (2005b) appointed participants of a larger sample to two subgroups according to their score in the STAI-T questionnaire [high trait anxious (HTA) and low trait anxious (LTA)] and found differential response patterns for these two subgroups. Therefore we took a similar approach by applying a median split to our sample according to the STAI-T score. Together with the within-subject factor condition (threat/safe) we included subgroup (HTA/LTA) as a between-subject factor into our analysis [2 × 2 factorial repeated measures analysis of variance (ANOVA)]. We also performed *post hoc t*-tests on the dependent variable (i.e., RTs) separately for both subgroups. Additionally, we used correlation analysis to quantify the association between the STAI-T score and the mean RT difference between threat blocks as compared to safe blocks in the whole sample. For all statistical tests, a level of significance *p* < 0.05 was used. Behavioral data were analyzed using SPSS Statistics 23 (IBM SPSS Statistics, Armonk, NY, USA).

### MRI Data-Acquisition

Images were acquired on a 3-Tesla Siemens Tim Trio scanner with the Siemens 32-channel head coil (Siemens, Erlangen, Germany). T1-weighted images were acquired with a 3D magnetization-prepared rapid gradient echo (MP-RAGE) sequence [repetition time (TR) = 1.9 s, echo time (TE) = 2.26 s, field of view (FOV) = 256 × 224 × 176 mm^3^, voxel size = 1 × 1 × 1 mm^3^, inversion time = 0.9 s, flip angle (FA) = 9°, phase partial Fourier 7/8, bandwidth (BW) = 200 Hz/Px]. Functional data was acquired with a multi-echo echo-planar imaging (EPI) research sequence with six echoes [Posse et al., 1999; TR = 2.54 s, TE = 8.6, 18.3, 28, 38, 48, and 57 ms, FOV = 192 × 192 × 132 mm^3^, voxel size = 4 × 4 × 3.2 mm^3^ with a slice gap of 25%, GRAPPA with ipat factor 3 and 42 reference lines, FA = 82°, BW = 2084 Hz/Px, slice orientation axial > coronal [A > C], slice order: descending]. EPI images were distortion-corrected in real-time using point spread function data from a dedicated sequence (parameters as for EPI, except TE = 8.7 ms; Zaitsev et al., 2004). Multi-echo images were combined using TE-dependent weights (0.59, 0.90, 1, 0.97, 0.88, 0.77), which are optimal for a T2^∗^-value of 30 ms (Posse et al., 1999).

### Data Analysis—Neuroimaging

#### Preprocessing

The fMRI data were preprocessed using statistical parametric mapping (SPM8, Wellcome Trust Centre for Neuroimaging, University College London, London, UK^1^) including slice-time correction to the middle slice, spatial realignment (motion correction), T1-based normalization, and smoothing with an isotropic Gaussian filter, 8 mm at full width at half maximum (FWHM) kernel (Penny et al., 2011). Functional data were resampled to 2 × 2 × 2 mm^3^ voxel size.

#### Correction of fMRI Data for Physiological Noise

Because neuroimaging data of the amygdala is potentially confounded by physiological noise such as cardiac and respiratory fluctuations which primarily affect cerebral vessels and cerebrospinal fluid and cause artifacts mimicking amygdala activation (Boubela et al., 2015), analysis of neuroimaging data was performed after correcting for physiological noise (for further information on results without correction, see Supplementary Material).

Physiological parameters were recorded via pulse oximetry on the middle finger of the left hand and a respiratory belt (peripheral equipment of the scanner). Correction for physiological noise was performed via an extension of the RETROspective Image CORrection (RETROICOR) approach (Glover et al., 2000; Hutton et al., 2011) using Fourier expansions of different order for the estimated phases of cardiac pulsation (third order), respiration (fourth order), and cardio-respiratory interactions (first order) (Hutton et al., 2011): the corresponding confound regressors were created using the Matlab physIO toolbox^2^ (Kasper et al., 2009). The 18 regressors for each subject (six cardiac, eight respiratory, four interaction regressors cardiac × respiratory) were entered into the general linear model (GLM) at the subject-level. Six motion regressors acquired during realignment were included.

#### First-Level Analysis

For subject-level statistical analysis, the GLM as implemented in SPM8 and a high-pass filter at 1/128 Hz (Penny et al., 2011) was used. Neural model components were convolved with SPMs canonical hemodynamic response function (HRF). Two separate approaches were employed [Neuroimaging Model 1 (Hypothesis 2) and Neuroimaging Model 2 (Hypothesis 3), see below].

##### Model 1 [effect of condition on amygdala BOLD signal (Hypothesis 2)]

Five conditions (predictor variables) were entered into the design matrix as separate regressors. Threat-blocks and shock-blocks were modeled as 30 s blocks. Safe-block onsets, threat-block onsets and shock-events were modeled as events. The safe condition (safe-blocks) served as the implicit baseline.

##### Model 2 [correlation of the BOLD signal in the amygdala prior to a CRT trial with the trial-specific RT (Hypothesis 3)]

The rationale of the model was to test whether neuronal activity in the amygdala prior to a task is correlated with the corresponding RT on a trial-by-trial basis irrespective of condition (threat/safe). First, we defined a regressor model that was not convolved with the HRF (see **Figure 4A**). We did not base our predictive claims (i.e., signals prior to task execution are correlated with performance) on a convolution approach, because, due to the extent of the HRF, we cannot exclude that task related signals are contaminating the resulting regression coefficient. The regressor model investigated the BOLD signal directly without making any assumptions about the neurovascular coupling. It clearly investigated the BOLD signal from one TR (∼2.5 s) before task onset to one TR (∼2.5 s) after task onset (rounded down to integer TRs), which cannot be contaminated by the BOLD response of the following task. In this model, two regressors are included: a constant regressor and a mean-free regressor proportional to the trial-specific RT. Both regressors comprised 285 TRs (data points) with each modeled event two TRs wide. Events were defined for each of the 48 RT trials except for shock trials and the first trial in the experiment (see Data Analysis—Behavioral). The model also included one separate nuisance regressor for the five shock events. We did not include blocks or block onsets (see Supplementary Material). To confirm the results of the regressor model *post hoc*, data analysis was repeated with a neural model. This model assumed parametrically modulated neural events prior to task onset that are convolved with the canonical HRF in SPM (Model 3; see Supplementary Material).

#### Second-Level Analysis

At the group-level, random-effects analysis was performed on the summary measures (i.e., regression coefficients). The contrast mean-free regressor vs. baseline was estimated. To assess effects of STAI-T subgroup (HTA/LTA) at a neuronal level subgroup assignment was additionally included as covariate and between-subject factor for group-level analysis. For region of interest (ROI) analysis on the group-level, we used SPM’s small volume correction (SVC). The ROI was defined by a mask for the bilateral amygdala created with the WFU PickAtlas (ANSIR Laboratory, WFU School of Medicine, Winston-Salem, NC, USA; left amygdala: 161 voxels, right amygdala: 158 voxels; also see Kobiella et al., 2011).

## Results

### Questionnaires

The mean results of the STAI, PSS, and BDI were within the range of 0.5 standard deviations of the normative data of a representative healthy population (Cohen, 1988; Whisman and Richardson, 2015). An overview is provided in **Supplementary Table S1**. Including the PSS and BDI in addition to the STAI served to address the consistency of the participants’ answers. In line with previous studies (Endler et al., 1992; Lee, 2012; Isobe et al., 2014), we found a correlation of the STAI-T score with the BDI-scores (*r* = 0.393, *p* = 0.008) and the PSS-scores (*r* = 0.493, *p* = 0.001; also see Supplementary Material for more detailed results and discussion).

### Evaluation of Threat Conditioning

A repeated measure ANOVA with the within-subject factor condition (threat/safe) and the between-subject factor STAI-T subgroup (HTA/LTA) was performed on the individual scores of the anxiety ratings (dependent variable; mean score for HTA in threat condition: 0.64 and safe condition: 0.05, mean score for LTA in threat condition: 0.57 and safe condition: 0.39). Eighteen participants indicated they were more anxious in the threat condition, 25 participants noted no difference in anxiety between conditions and two participants stated that they were more anxious during the presentation of the safe screen. We found a significant subgroup × condition interaction [*F*(1,43) = 5.8, *p* = 0.021] on anxiety ratings. There was a main effect of condition [*F*(1,43) = 19.3, *p* < 0.000]. There was no main effect of subgroup [*F*(1,43) = 0.9, *p* = 0.343]. *Post hoc* two-tailed paired samples *t*-tests revealed that only the subgroup of high anxious participants retrospectively reported higher anxiety in the threat condition compared to the safe condition [*t*(21) = 4.695, *p* < 0.000] and that only a marginal difference of the experience of anxiety between conditions was present for the subgroup of low anxious participants [*t*(22) = 1.447, *p*_one-tailed_ = 0.081].

### Effect of Condition on RTs (Hypothesis 1)

An overview of mean and median RTs of the two conditions (threat/safe) is displayed for the whole sample and for the two STAI-T subgroups in **Table 1**.

**Table 1:** Behavioral results of the CRT task.

Whole sample (*N* = 45)			High trait anxious subgroup (*N* = 22)		Low trait anxious subgroup (*N* = 23)	
			
Condition	RT (ms)	SE (ms)	RT (ms)	SE (ms)	RT (ms)	SE (ms)
**Danger**					
Mean	432	11	426	15	437	15
Median	420	11	414	16	426	15
**Safe**					
Mean	437	11	442	18	432	13
Median	424	10	429	15	420	12
**Δ Danger–safe**					
Mean Δ of mean	-5	4	-16^a^	5	5	5
Mean Δ of median	-4	4	-15^a^	6	6	6


*Results are presented separately in columns for the whole sample, for the subgroup of high-trait anxious individuals and for the subgroup of low-trait anxious individuals. For each group, mean and median reaction times (RTs) for the threat condition and the safe condition as well as the difference of mean and median RTs between conditions are presented.*

*^a^*P*_*two-tailed*_ < 0.05.*

**FIGURE 2:**
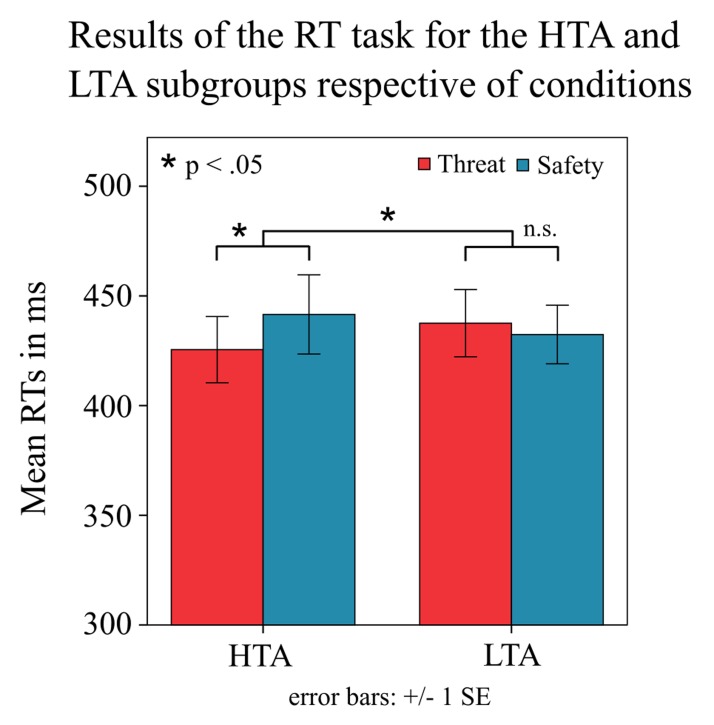
**Behavioral results by subgroup.** The plot shows the mean reaction times (RTs) with standard errors (SE) for the different conditions (threat/safe) in the subgroup of high-trait anxious (HTA) and low-trait anxious individuals (LTA). Subgroups differed in the difference in RTs between conditions. The HTA subgroup showed faster responses in the threat condition compared to the safe condition.

A 2 × 2 factorial repeated measures ANOVA with the within-subject factor condition and the between-subject factor subgroup revealed a significant condition × subgroup interaction [means: *F*(1,43) = 9.4, *p* = 0.004, $\eta_{p}^{2}$ = 0.180; medians: *F*(1,43) = 6.1, *p* = 0.018, $\eta_{p}^{2}$ = 0.123; **Figure 2**]. There was no main effect of condition [means: *F*(1,43) = 2.5, *p* = 0.123; medians: *F*(1,43) = 1.1, *p* = 0.307] and no main effect of subgroup [means: *F*(1,43) = 0, *p* = 0.947; medians: *F*(1,43) = 0, *p* = 0.954]. As expected, *post hoc* analysis (two-tailed paired samples *t*-test) showed that the high-anxious subgroup had faster RTs during the threat condition compared to the safe condition [means: *t*(21) = -3.076, *p* = 0.006; medians: *t*(21) = -2.455, *p* = 0.023; **Figure 2**]. However, in the low-anxious subgroup there was no effect of condition on RTs [means: *t*(22) = 1.135, *p* = 0.269; medians: *t*(22) = 1.009, *p* = 0.324].

**FIGURE 3:**
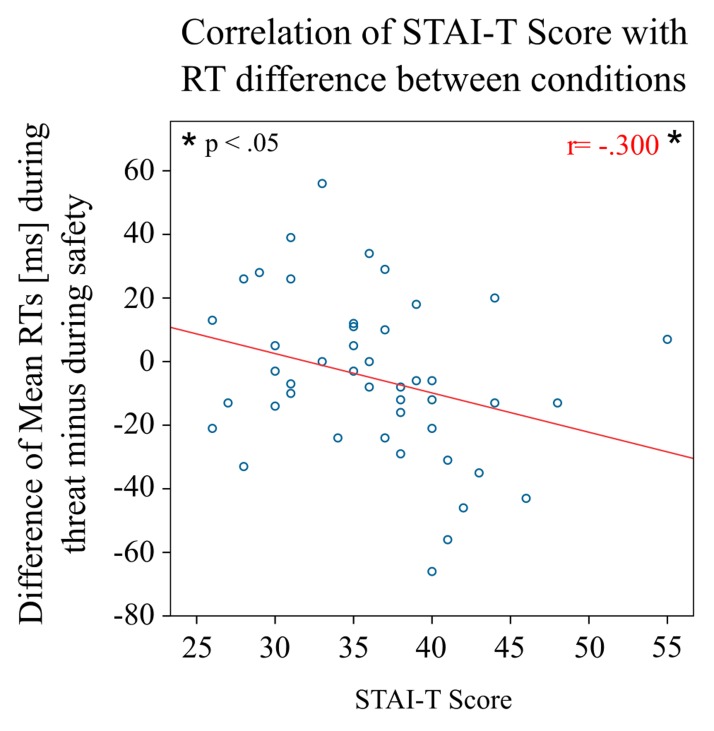
**Behavioral results by individual STAI-T scores.** The plot shows the mean difference in reaction times in the threat condition compared to the safe condition for individual participants with respect to their score in the STAI-T questionnaire. The higher the individual score on trait anxiety, the faster the participant responded in the threat condition compared to the safe condition.

In line with this finding, there was a negative correlation of the STAI-T score with the mean RT difference between threat blocks as compared to safe blocks (*r* = -0.300, *p* = 0.045), that is, the higher the participants scored on trait anxiety, the faster were their RTs in the threat condition as compared to the safe condition (**Figure 3**). No such correlation was found separately for one of the two subgroups (both *p* > 0.4).

Independent samples *t*-tests showed that there was neither a significant difference in RTs in the danger condition nor in RTs in the safe condition between the two subgroups (all *p* > 0.4). Misses were rare (*M* = 2.4, *SD* = 4.9) and did not differ significantly between conditions [*t*(44) = 1.356, *p* = 0.182].

### Effect of Condition on Amygdala BOLD Signal (Hypothesis 2)

The analysis of the neuroimaging data did not reveal a main effect of condition (threat/safe) on amygdala activity [SVC at uncorrected *p* < 0.01, extent threshold k (KE) ≥ 8], neither in the whole sample nor within the two STAI-T subgroups (HTA/LTA; Model 1, see Materials and Methods). More precisely, there was no significant difference of the BOLD response within the bilateral amygdala, neither between the threat and safe block conditions nor between the threat and safe block onsets. However, when including subgroup as a covariate, we noted a marginal increase of activation within the left amygdala in threat-blocks in contrast to safe-blocks (SVC, Montreal Neurological Institute (MNI): -30/-2/-22, family-wise error, FWE_corr_ *p* = 0.083, KE ≥ 0) for high trait-anxiety compared to low trait-anxiety. This finding was not confirmed for block onsets.

### Correlation of the BOLD Signal in the Amygdala Prior to a CRT Trial with the Trial-Specific RT (Hypothesis 3)

**FIGURE 4:**
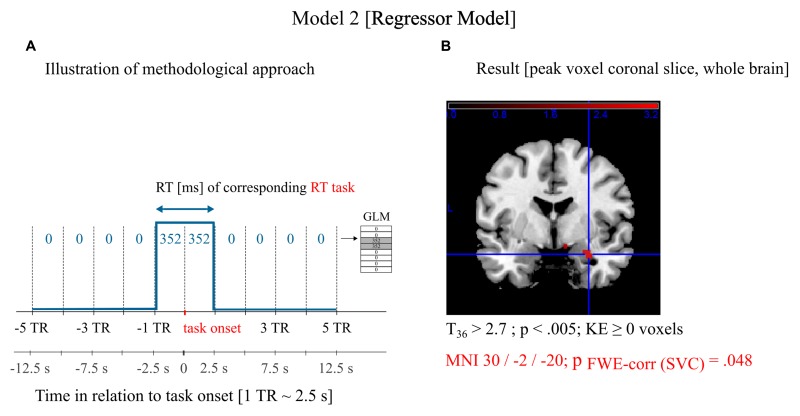
**Neuroimaging data analysis and results.** **(A)** Graphical illustration of the first-level model. The regressor model was not convolved with the HRF and investigated the BOLD signal directly to address correlations of the neuronal activity *prior* to a CRT trial with the trial-specific RT (Hypothesis 3). The experiment had 285 repetition times (285 data points). For each TR prior to and at task onset the respective reaction time (RT) of that trial was included as data point. All other data points were 0. **(B)** Results of the second-level analysis. BOLD-signal within the right amygdala around the RT task correlated with the RT of the corresponding trial. The anatomical illustration shows a coronal slice overlaid with the *t*-map at whole brain level. The result of the statistical analysis is presented after small volume correction (SVC).

SPM ROI analysis (Model 2; see Materials and Methods) revealed a significant positive trial-by-trial correlation between BOLD activation within the right amygdala prior to the task and subsequent RTs irrespective of condition (threat/safe): MNI: 30/-2/-20; FWE_corr_ *p* = 0.048 (**Figure 4B**). We confirmed *post hoc* that the results are consistent with a neural model (Model 3; see Supplementary Material): SVC, MNI: 30/-2/-22; FWE_corr_ *p* = 0.012 (**Supplementary Figure S1**). The covariate subgroup (HTA/LTA) did not reveal significant differences in BOLD-RT correlation in the right amygdala between subsamples (SVC, uncorrected *p* > 0.01). Independent two-sample *t*-test revealed no significant differences in the defined contrast between subgroups after SVC.

## Discussion

In line with the literature, the current study showed that trait anxiety affects performance in a simple CRT task during exposure to continuous threat. Critically, the study additionally revealed amygdala activity as a potential predictor of RTs in a threatening environment. Higher BOLD signal within the right amygdala prior to a CRT task was related to longer RTs on the respective trial. Unexpectedly, the design failed to show a general increase in amygdala activation in response to threat compared to a control condition.

Our first hypothesis predicted that, after threat conditioning, RTs in a CRT task would be faster in the threat compared to the safe condition in individuals with higher trait anxiety (Koster et al., 2005b). This hypothesis was confirmed; more anxious participants responded faster in the threat compared to the safe condition, while less anxious participants did not. This pattern is compatible with past research. First, high anxious participants are more sensitive to threat conditioning than healthy controls (see Lissek et al., 2005 for a meta-analysis) as also indicated by the state anxiety ratings in our study. High anxious participants reported higher anxiety in the threat condition compared to the safe condition at the end of the experiment. However, retrospective rating of anxiety might also be biased by enhanced memory for threat in more anxious individuals (Reidy and Richards, 1997; McCabe, 1999). Second, responses to threat are more pronounced with increased trait anxiety in terms of early sensory processing (Weinstein, 1995), physiological reactivity (Najstrom and Jansson, 2007), attention (Yiend and Mathews, 2001), and behavior (Onnis et al., 2011). Additionally, there is no evidence in our data that the effect of faster RTs during threat in the high-anxious sample could be accounted for by depressive mood, which is often associated with higher anxiety (see Supplementary Material). Note that the association of trait anxiety and sensitivity to threat conditioning in our data does not allow disentangling which of the two caused the observed RT difference in the high-anxious subgroup.

In our second hypothesis, we predicted that neuronal activity in the amygdala is increased during a threat compared to a safe condition. Threat conditioning has been demonstrated in the past to be very effective in pairing an US with a CS (see LeDoux, 2014 for a review). Also, the amygdala has been shown to respond to a threat CS (see Phelps and LeDoux, 2005 for a review). The current experiment was close to reveal higher amygdala activation during the exposure to threat in the high trait-anxiety subgroup (FWE_corr_ *p* = 0.083). Although not significant, this trend indicates the presence of some neuronal correlate for the behavioral differences in subgroups (Hypothesis 1). We did not find a robust effect on the amygdala BOLD signal contrast between conditions, that is, exposure to the threat of a potential electric shock compared to a safe control condition. This result was surprising and possibly due to the following four factors:

(1) Effectiveness of threat conditioning was limited. Participants in the low-anxious subgroup did not report statistically different anxiety ratings for the two conditions. Because anxiety ratings were moderate overall, we do not know whether the design was insufficient to induce threat or whether the background images used for conditioning were difficult to distinguish. However, we found an RT difference between conditions in this study for the high-anxious subgroup and marginally also in a pilot study for a separate, smaller sample of participants [means: *t*(11) = 2.2, *p* = 0.050)]. Both findings indicate a fair success of conditioning. Nevertheless, we are missing a clear indicator of the effectiveness of conditioning given that both amygdala BOLD signal and skin conductance (in a pilot study) did not show such an effect with sufficient confidence.
(2) Habituation over time (Fischer et al., 2003) may have diminished block effects as the threat screen was presented continuously for 30 s, which is a much longer CS presentation than in more conventional conditioning studies (Choi et al., 2012; Schmidt et al., 2015). To address this point, we included event-related conditions in our analysis to detect a difference in amygdala activation to imminent changes of condition (threat/safe) at block onsets. But no significant statistical effect was detected potentially due to the low number of events (i.e., 12 per condition).
(3) Specific functionalities of different brain regions in the processing of threat have to be considered. While the amygdala is activated by unpredictable, imminent threat, an extended network including, for example, the BNST, the hippocampus, the vmPFC and the insula, is more specifically involved in processing predictable, sustained threat (i.e., hazardous environment; Walker et al., 2003; Sullivan et al., 2004; Kalisch et al., 2006; Alvarez et al., 2011; Andreatta et al., 2015; Herrmann et al., 2016). In addition, Andreatta et al. (2015) discussed primary motor cortex activation during sustained fear as representing participants’ desire to leave a virtual reality associated with threat. Unfortunately, a ROI-analysis of the BNST response to sustained threat could not be performed, because the nucleus is very small, not easily identifiable by an anatomical atlas, and the current design did not offer a localizer (Somerville et al., 2010). ROI-analysis for the bilateral insula yielded no significant results (see Supplementary Material). This was expected, because although the insula has been suggested in sustained threat processing (Paulus and Stein, 2006; Herrmann et al., 2016), the region has been shown to predominantly increase activity at stimulus onset and also to co-vary with activation within the amygdala (Carlson et al., 2011). It is tempting to consider the motor cortex as having a specific function (Andreatta et al., 2015), for example, concerning the whole-body response to sustained threat. Therefore a potential effect on RTs is easy to imagine. A ROI-analysis within the current project, however, would have been explorative, but should be addressed in future work. ROI-analysis for hippocampus and vmPFC seemed not appropriate (see Supplementary Material). Nevertheless, it is reasonable to assume that faster RTs during threat in HTA individuals are reflected on the neurofunctional level by a network of different brain regions. Activation of these nodes, however, is likely to be quantitatively different between individuals. Multi-voxel pattern analysis (Haynes, 2015) may deliver higher sensitivity to test this assumption in future experiments.
(4) Because the task used in this study was effortful rather than automatic in nature, a negative coupling of brain regions involved in cognitive control (e.g., the prefrontal cortex) with limbic regions might have caused an attenuation of amygdala neuronal activity (Banks et al., 2007; Gyurak et al., 2011; Gold et al., 2015).

Extinction after the conditioning phase, on the other hand, is unlikely an explanation for two reasons: the amygdala is also involved in early threat extinction (LaBar et al., 1998; Phelps et al., 2004) and electric shocks have also been applied during certain trials of the CRT task (which were excluded in the analyzed contrast).

We suggest that, on a neuronal level, spontaneous fluctuations in amygdala fMRI signal in a threat-laden environment rather than block-related changes shape behavior in the current experimental design. In line with this suggestion, in our third hypothesis, we predicted that BOLD signal in the amygdala prior to a task is correlated with the corresponding RTs on a trial-by-trial basis irrespective of condition. Our third hypothesis was confirmed. Statistical analysis showed that the higher the amygdala activity before a task, the slower the participant’s response was in that trial. The reported BOLD-RT correlation was found in a regressor model; therefore we could exclude that task related signals due to the extent of the HRF affected the results. We confirmed *post hoc* that the results of the regressor model can be reproduced with convolved “neural” regressors.

Threat in an evolutionary perspective should mobilize resources (i.e., fight or flight; Cannon, 1915) to enhance response to threat. Here, we found faster responses to a RT cue in the threat condition and a trend to higher amygdala activation in the threat blocks for the high-anxious subgroup. Given this, the positive BOLD-RT correlation in this study might not be intuitive and needs further reflection. Threat captures attention. For example, dot-probe tasks nicely show that it is more difficult to spatially detach from a threat stimulus compared to a neutral stimulus. RTs to a cue are often faster when the cue is presented at the position of a previous threat-laden stimulus compared to when presented at a different position (Frewen et al., 2008). The same effect was observed in another task within the broader research project (manuscript in preparation). We found slower responses in a RT task if the cue was preceded by a negative image that captured attention. Although speculative, in this study a continuous awareness of threat (not necessarily with respect to condition) might have drawn cognitive resources and resulted in overall slower responses. This explanation would be in line with the observed positive BOLD–RT correlation. Why RTs were faster during the threat condition in the high-anxious subgroup with regard to this explanation unfortunately remains an open question, especially because neuroimaging results revealed no significant difference between subgroups.

Correction for cardiac and respiratory artifacts (Boubela et al., 2015) was applied. Venous artifacts by the basal vein of Rosenthal, (i) which drains the fusiform face area and (ii) which has been discussed to potentially confound fMRI amygdala signal (Mende-Siedlecki et al., 2013; Boubela et al., 2015), is unlikely, because face stimuli were not used in the current design. Hence, even though physiological noise can never be entirely discarded to confound fMRI results, it can be deemed unlikely in the current study. Note that on the other hand, physiological noise correction reduces the desired signal by reduction of variance, if physiological noise is synchronous with task activation (Glover et al., 2000). This is true for experimental designs that assess emotional processing (Lipp et al., 2014), where arousal is reflected in cardiac (Kusserow et al., 2013; Azarbarzin et al., 2014) or/and respiratory measures (Vlemincx et al., 2015). Results of an additional analysis without physiological corrections confirm a reduction of statistical power by physiological noise correction in the current study (see Supplementary Material).

However, we found a significant trial-by-trial correlation of amygdala fMRI signal prior to the CRT task with the RT. Hence, we propose that the amygdala fMRI signal predicts behavioral performance in a threat-laden environment; although, the result has to be interpreted with caution (FWE_corr_ *p* = 0.048) until reproduced by an independent experiment. It is very possible that the current design favored the detection of such an effect due to the exposure to threat and safe blocks and might not be detectable under non-threatening conditions. On the other hand, the effect could also not be associated with threat processing specifically. Claims about the latter assumption cannot be made based on the current design and need to be addressed in further research. We show in the Supplementary Material that the variance of the data is further reduced by including blocks and block onsets and that the reported effect is then no longer significant.

In conclusion, the current study successfully identified factors that explain variability of RTs observed in past research. The results highlight an influence of personality trait on the modulation of task performance by experimental factors such as threat. We show that inter-individual variability in RTs during threat exposure can be partially explained by inter-individual differences in trait anxiety of healthy adults. Critically, results additionally show that on a trial-by-trial basis amygdala fMRI signal prior to a task predicts RTs when participants are exposed to threat. This finding is important in two ways: first, it shows a trial-by-trial influence of amygdala activity on behavior rather than correlations of mean activity and mean task performance across subjects. Second, spontaneous activity fluctuations in the amygdala are likely to drive these changes in behavior.

The current design with repeated background changes, unjittered RT task onsets and shock events potentially affecting baseline variance can be further developed to investigate the circumstances in which amygdala fMRI signal is a sensitive predictor of RTs. To avoid circular analysis (Kriegeskorte et al., 2009), the effect size (i.e., predictive value) of the detected BOLD-RT correlation was not calculated here and needs to be estimated in a second independent study. Further studies also need to be conducted to reveal why, how, and in what temporal window prior to a task, the amygdala activation affects behavioral performance. Increased monitoring of the environment with increased activation of the amygdala and therefore a less focused deployment of cognitive resources toward the task could be one speculative explanation for longer RTs.

## Author Contributions

MJ, DM, NV, MS, and MM contributed to the experimental design of the study. Data acquisition was carried out by MJ and MM. Data analysis was performed by PR, DM, and MM. PR, MJ, MS, and MM were involved in the interpretation of data. The manuscript was drafted by PR and MM. All authors (PR, MJ, DM, NV, MS, and MM) revised the manuscript critically, approved the submitted version to be published, and hold themselves accountable for all aspects of the work in ensuring that questions related to the accuracy or integrity of any part of the work are appropriately investigated and resolved.

## Conflict of Interest Statement

The authors declare that the research was conducted in the absence of any commercial or financial relationships that could be construed as a potential conflict of interest.

## Footnotes

1 http://www.fil.ion.ucl.ac.uk/spm

2 http://www.translationalneuromodeling.org/team/lars-kasper/

## References

[B1] Alvarez R. P. Chen G. Bodurka J. Kaplan R. Grillon C. (2011). Phasic and sustained fear in humans elicits distinct patterns of brain activity. *Neuroimage* 55 389–400. 10.1016/j.neuroimage.2010.11.057 21111828 PMC3100535

[B2] Ando S. Kida N. Oda S. (2004). Retention of practice effects on simple reaction time for peripheral and central visual fields. *Percept. Mot. Skills* 98 897–900. 10.2466/pms.98.3.897-900 15209305

[B3] Andreatta M. Glotzbach-Schoon E. Muhlberger A. Schulz S. M. Wiemer J. Pauli P. (2015). Initial and sustained brain responses to contextual conditioned anxiety in humans. *Cortex* 63 352–363. 10.1016/j.cortex.2014.09.014 25460498

[B4] Azarbarzin A. Ostrowski M. Hanly P. Younes M. (2014). Relationship between arousal intensity and heart rate response to arousal. *Sleep* 37 645–653. 10.5665/sleep.3560 24899756 PMC4044744

[B5] Bach D. R. Hurlemann R. Dolan R. J. (2015). Impaired threat prioritisation after selective bilateral amygdala lesions. *Cortex* 63 206–213. 10.1016/j.cortex.2014.08.017 25282058 PMC4317193

[B6] Banks S. J. Eddy K. T. Angstadt M. Nathan P. J. Phan K. L. (2007). Amygdala-frontal connectivity during emotion regulation. *Soc. Cogn. Affect. Neurosci.* 2 303–312. 10.1093/scan/nsm029 18985136 PMC2566753

[B7] Baur V. Hanggi J. Jancke L. (2012). Volumetric associations between uncinate fasciculus, amygdala, and trait anxiety. *BMC Neurosci* 13:4 10.1186/1471-2202-13-4 PMC3398321 22217209

[B8] Beck A. T. Steer R. A. Ball R. Ranieri W. F. (1996). Comparison of beck depression inventories-IA and -II in psychiatric outpatients. *J. Pers. Assess.* 67 588–597. 10.1207/s15327752jpa6703_13 8991972

[B9] Bishop D. T. Karageorghis C. I. Kinrade N. P. (2009). Effects of musically-induced emotions on choice reaction time performance. *Sport Psychol.* 23 59–76. 10.1123/tsp.23.1.59

[B10] Bishop S. Duncan J. Lawrence A. D. (2004). Prefrontal cortical function and anxiety: controlling attention to threat-related stimuli. *Nat. Neurosci.* 7 184–188. 10.1038/nn1173 14703573

[B11] Blair K. S. Smith B. W. Mitchell D. G. V. Morton J. Vythilingam M. Pessoa L. (2007). Modulation of emotion by cognition and cognition by emotion. *Neuroimage* 35 430–440. 10.1016/j.neuroimage.2006.11.048 17239620 PMC1862681

[B12] Boubela R. N. Kalcher K. Huf W. Seidel E. M. Derntl B. Pezawas L. (2015). fMRI measurements of amygdala activation are confounded by stimulus correlated signal fluctuation in nearby veins draining distant brain regions. *Sci. Rep*, 5 10499 10.1038/Srep10499 PMC4440210 25994551

[B13] Buchsbaum M. Callaway E. (1965). Influences of respiratory cycle on simple reaction time. *Percept. Mot. Skills* 20 961–966. 10.2466/pms.1965.20.3.961 14314021

[B14] Bugg J. M. Jacoby L. L. Toth J. P. (2008). Multiple levels of control in the Stroop task. *Mem. Cognit.* 36 1484–1494. 10.3758/MC.36.8.1484 PMC2682765 19015507

[B15] Buodo G. Sarlo M. Palomba D. (2002). Attentional resources measured by reaction times highlight differences within pleasant and unpleasant, high arousing stimuli. *Mot. Emot.* 26 123–138. 10.1023/A:1019886501965

[B16] Cannon W. B. (1915). *Bodily Changes in Pain, Hunger, Fear, and Rage: An Account of Recent Researches Into the Function of Emotional Excitement*. New York, NY: D. Appleton and company.

[B17] Carlson J. M. Greenberg T. Rubin D. Mujica-Parodi L. R. (2011). Feeling anxious: anticipatory amygdalo-insular response predicts the feeling of anxious anticipation. *Soc. Cogn. Affect. Neurosci.* 6 74–81. 10.1093/scan/nsq017 20207692 PMC3023082

[B18] Choi J. M. Padmala S. Pessoa L. (2012). Impact of state anxiety on the interaction between threat monitoring and cognition. *Neuroimage* 59 1912–1923. 10.1016/j.neuroimage.2011.08.102 21939773 PMC3230669

[B19] Cohen S. (1988). “Perceived stress in a probability sample of the United States,” in *The Social Psychology of Health*, eds Spacapam S. Oskamp S. (Thousand Oaks, CA: Sage Publications, Inc), 251.

[B20] Cohen S. Kamarck T. Mermelstein R. (1983). A global measure of perceived stress. *J. Health Soc. Behav.* 24 385–396. 10.2307/2136404 6668417

[B21] Der G. Deary I. J. (2006). Age and sex differences in reaction time in adulthood: results from the United Kingdom health and lifestyle survey. *Psychol. Aging* 21 62–73. 10.1037/0882-7974.21.1.62 16594792

[B22] Dumas T. Dubal S. Attal Y. Chupin M. Jouvent R. Morel S. (2013). MEG evidence for dynamic amygdala modulations by gaze and facial emotions. *PLoS ONE* 8:e74145 10.1371/journal.pone.0074145 PMC3769395 24040190

[B23] Eason R. G. Harter M. R. White C. T. (1969). Effects of attention and arousal on visually evoked cortical potentials and reaction time in man. *Physiol. Behav.* 4 283–289. 10.1016/0031-9384(69)90176-0

[B24] Eden A. S. Schreiber J. Anwander A. Keuper K. Laeger I. Zwanzger P. (2015). Emotion regulation and trait anxiety are predicted by the microstructure of fibers between amygdala and prefrontal cortex. *J. Neurosci.* 35 6020–6027. 10.1523/Jneurosci.3659-14.2015 25878275 PMC6605169

[B25] Endler N. S. Cox B. J. Parker J. D. Bagby R. M. (1992). Self-reports of depression and state-trait anxiety: evidence for differential assessment. *J. Pers. Soc. Psychol.* 63 832–838. 10.1037/0022-3514.63.5.832 1447695

[B26] Etkin A. Klemenhagen K. C. Dudman J. T. Rogan M. T. Hen R. Kandel E. R. (2004). Individual differences in trait anxiety predict the response of the basolateral amygdala to unconsciously processed fearful faces. *Neuron* 44 1043–1055. 10.1016/j.neuron.2004.12.006 15603746

[B27] Farber I. E. Spence K. W. (1956). Effects of anxiety, stress, and task variables on reaction-time. *J. Pers.* 25 1–18. 10.1111/j.1467-6494.1956.tb01284.x 13368028

[B28] Fernandes O. Portugal L. C. L. Alves R. C. S. Campagnoli R. R. Mocaiber I. David I. P. A. (2013). How you perceive threat determines your behavior. *Front. Hum. Neurosci.* 7:632 10.3389/fnhum.2013.00632 PMC3792557 24115925

[B29] Fischer H. Wright C. I. Whalen P. J. McInerney S. C. Shin L. M. Rauch S. L. (2003). Brain habituation during repeated exposure to fearful and neutral faces: a functional MRI study. *Brain Res. Bull.* 59 387–392. 10.1016/S0361-9230(02)00940-1 12507690

[B30] Fox E. Russo R. Bowles R. Dutton K. (2001). Do threatening stimuli draw or hold visual attention in subclinical anxiety? *J. Exp. Psychol. Gen.* 130 681–700. 10.1037/0096-3445.130.4.681 11757875 PMC1924776

[B31] Frewen P. A. Dozois D. J. A. Joanisse M. F. Neufeld R. W. J. (2008). Selective attention to threat versus reward: meta-analysis and neural-network modeling of the dot-probe task. *Clin. Psychol. Rev.* 28 307–337. 10.1016/j.cpr.2007.05.006 17618023

[B32] Glover G. H. Li T. Q. Ress D. (2000). Image-based method for retrospective correction of physiological motion effects in fMRI: RETROICOR. *Magn. Reson. Med.* 44 162–167. 10.1002/1522-2594(200007)44:1<162::AID-MRM23>3.3.CO;2-5 10893535

[B33] Gold A. L. Morey R. A. McCarthy G. (2015). Amygdala-prefrontal cortex functional connectivity during threat-induced anxiety and goal distraction. *Biol. Psychiatry* 77 394–403. 10.1016/j.biopsych.2014.03.030 24882566 PMC4349396

[B34] Greening S. G. Mitchell D. G. (2015). A network of amygdala connections predict individual differences in trait anxiety. *Hum. Brain Mapp.* 36 4819–4830. 10.1002/hbm.22952 26769550 PMC6869108

[B35] Gyurak A. Gross J. J. Etkin A. (2011). Explicit and implicit emotion regulation: a dual-process framework. *Cogn. Emot.* 25 400–412. 10.1080/02699931.2010.544160 21432682 PMC3280343

[B36] Haynes J. D. (2015). A primer on pattern-based approaches to fMRI: principles, pitfalls, and perspectives. *Neuron* 87 257–270. 10.1016/j.neuron.2015.05.025 26182413

[B37] Herrmann M. J. Boehme S. Becker M. P. Tupak S. V. Guhn A. Schmidt B. (2016). Phasic and sustained brain responses in the amygdala and the bed nucleus of the stria terminalis during threat anticipation. *Hum. Brain Mapp.* 37 1091–1102. 10.1002/hbm.23088 26678871 PMC6867277

[B38] Hutton C. Josephs O. Stadler J. Featherstone E. Reid A. Speck O. (2011). The impact of physiological noise correction on fMRI at 7 T. *Neuroimage* 57 101–112. 10.1016/j.neuroimage.2011.04.018 21515386 PMC3115139

[B39] Isobe K. Ishizu T. Oikawa H. Kamimaki T. Ishijima M. Nanmoku T. (2014). Correlation between blood biomarkers and depression and anxiety scales in apparently healthy individuals. *Int. J. Anal. Bio-Sci.* 2 163–166.

[B40] Johanson A. M. (1922). The influence of incentive and punishment upon reaction-time. *Arch. Psychol.* 54 1–53.

[B41] Kaczkurkin A. N. Moore T. M. Ruparel K. Ciric R. Calkins M. E. Shinohara R. T. (2016). Elevated amygdala perfusion mediates developmental sex differences in trait anxiety. *Biol. Psychiatry* 10.1016/j.biopsych.2016.04.021 [Epub ahead of print]. PMC5074881 27395327

[B42] Kalisch R. Korenfeld E. Stephan K. E. Weiskopf N. Seymour B. Dolan R. J. (2006). Context-dependent human extinction memory is mediated by a ventromedial prefrontal and hippocampal network. *J. Neurosci.* 26 9503–9511. 10.1523/Jneurosci.2021-06.2006 16971534 PMC2634865

[B43] Kamin L. J. Clark J. W. (1957). The taylor scale and reaction-time. *J. Abnorm. Soc. Psychol.* 54 262–263. 10.1037/H0046413 13428461

[B44] Kappenman E. S. MacNamara A. Proudfit G. H. (2015). Electrocortical evidence for rapid allocation of attention to threat in the dot-probe task. *Soc. Cogn. Affect. Neurosci.* 10 577–583. 10.1093/scan/nsu098 25062842 PMC4381248

[B45] Kasper L. Marti S. Vannesjö S. Hutton C. Dolan R. Weiskopf N. (2009). Cardiac artefact correction for human brainstem fMRI at 7T. *Neuroimage* 47 S100 10.1016/S1053-8119(09)70854-7

[B46] Kendall P. C. Finch A. J. Auerbach S. M. Hooke J. F. Mikulka P. J. (1976). State-trait anxiety inventory – systematic evaluation. *J. Consult. Clin. Psychol.* 44 406–412. 10.1037/0022-006x.44.3.406 932270

[B47] Kirita T. Endo M. (1995). Happy face advantage in recognizing facial expressions. *Acta Psychol. (Amst.)* 89 149–163. 10.1016/0001-6918(94)00021-8

[B48] Kobiella A. Reimold M. Ulshofer D. E. Ikonomidou V. N. Vollmert C. Vollstadt-Klein S. (2011). How the serotonin transporter 5-HTTLPR polymorphism influences amygdala function: the roles of in vivo serotonin transporter expression and amygdala structure. *Transl. Psychiatry* 1:e37 10.1038/tp.2011.29 PMC3309509 22832611

[B49] Koster E. H. W. Crombez G. Van Damme S. Verschuere B. De Houwer J. (2005a). Signals for threat modulate attentional capture and holding: Fear-conditioning and extinction during the exogenous cueing task. *Cogn. Emot.* 19 771–780. 10.1080/02699930441000418

[B50] Koster E. H. W. Verschuere B. Crombez G. Van Damme S. (2005b). Time-course of attention for threatening pictures in high and low trait anxiety. *Behav. Res. Ther.* 43 1087–1098. 10.1016/j.brat.2004.08.004 15922291

[B51] Kriegeskorte N. Simmons W. K. Bellgowan P. S. Baker C. I. (2009). Circular analysis in systems neuroscience: the dangers of double dipping. *Nat. Neurosci.* 12 535–540. 10.1038/nn.2303 19396166 PMC2841687

[B52] Kusserow M. Amft O. Troster G. (2013). Modeling Arousal Phases in Daily Living Using Wearable Sensors. *IEEE Trans. Affect. Comput.* 4 93–105. 10.1109/T-Affc.2012.37

[B53] LaBar K. S. Gatenby J. C. Gore J. C. LeDoux J. E. Phelps E. A. (1998). Human amygdala activation during conditioned fear acquisition and extinction: a mixed-trial fMRI study. *Neuron* 20 937–945. 10.1016/S0896-6273(00)80475-4 9620698

[B54] Labar K. S. Ledoux J. E. Spencer D. D. Phelps E. A. (1995). Impaired fear conditioning following unilateral temporal lobectomy in humans. *J. Neurosci.* 15 6846–6855. 7472442 10.1523/JNEUROSCI.15-10-06846.1995 PMC6578018

[B55] Landauer A. A. Armstrong S. Digwood J. (1980). Sex difference in choice reaction-time. *Br. J. Psychol.* 71 551–555. 10.1111/j.2044-8295.1980.tb01766.x

[B56] LeDoux J. (2003). The emotional brain, fear, and the amygdala. *Cell. Mol. Neurobiol.* 23 727–738. 10.1023/A:1025048802629 14514027 PMC11530156

[B57] LeDoux J. E. (2014). Coming to terms with fear. *Proc. Natl. Acad. Sci. U.S.A.* 111 2871–2878. 10.1073/pnas.1400335111 24501122 PMC3939902

[B58] Lee E.-H. (2012). Review of the psychometric evidence of the perceived stress scale. *Asian Nurs. Res. (Korean Soc. Nurs. Sci.)* 6 121–127. 10.1016/j.anr.2012.08.004 25031113

[B59] Leppanen J. M. Tenhunen M. Hietanen J. K. (2003). Faster choice-reaction times to positive than to negative facial expressions – The role of cognitive and motor processes. *J. Psychophysiol.* 17 113–123. 10.1027/0269-8803.17.3.113

[B60] Lipp I. Murphy K. Wise R. G. Caseras X. (2014). Understanding the contribution of neural and physiological signal variation to the low repeatability of emotion-induced BOLD responses. *Neuroimage* 86 335–342. 10.1016/j.neuroimage.2013.10.015 24128735 PMC3898985

[B61] Lissek S. Powers A. S. McClure E. B. Phelps E. A. Woldehawariat G. Grillon C. (2005). Classical fear conditioning in the anxiety disorders: a meta-analysis. *Behav. Res. Ther.* 43 1391–1424. 10.1016/j.brat.2004.10.007 15885654

[B62] Lonsdorf T. B. Haaker J. Kalisch R. (2014). Long-term expression of human contextual fear and extinction memories involves amygdala, hippocampus and ventromedial prefrontal cortex: a reinstatement study in two independent samples. *Soc. Cogn. Affect. Neurosci.* 9 1973–1983. 10.1093/scan/nsu018 24493848 PMC4249485

[B63] Macleod C. M. (1991). Half a century of research on the stroop effect – an integrative review. *Psychol. Bull.* 109 163–203. 10.1037/0033-2909.109.2.163 2034749

[B64] Mama Y. Ben-Haim M. S. Algom D. (2013). When emotion does and does not impair performance: a Garner theory of the emotional Stroop effect. *Cogn. Emot.* 27 589–602. 10.1080/02699931.2012.726212 23025518

[B65] Marxen M. Jacob M. J. Muller D. K. Posse S. Ackley E. Hellrung L. (2016). Amygdala regulation following fmri-neurofeedback without instructed strategies. *Front. Hum. Neurosci.* 10:183 10.3389/fnhum.2016.00183 PMC4844623 27199706

[B66] McCabe R. E. (1999). Implicit and explicit memory for threat words in high- and low-anxiety-sensitive participants. *Cognit. Ther. Res.* 23 21–38. 10.1023/A:1018706607051

[B67] McDonald L. M. Griffin H. J. Angeli A. Torkamani M. Georgiev D. Jahanshahi M. (2015). Motivational modulation of self-initiated and externally triggered movement speed induced by threat of shock: experimental evidence for paradoxical kinesis in Parkinson’s disease. *PLoS ONE* 10:e0135149 10.1371/journal.pone.0135149 PMC4540447 26284366

[B68] Mende-Siedlecki P. Verosky S. C. Turk-Browne N. B. Todorov A. (2013). Robust selectivity for faces in the human amygdala in the absence of expressions. *J. Cogn. Neurosci.* 25 2086–2106. 10.1162/jocn_a_00469 23984945 PMC4029947

[B69] Mogg K. Bradley B. P. (1999). Orienting of attention to threatening facial expressions presented under conditions of restricted awareness. *Cogn. Emot.* 13 713–740. 10.1080/026999399379050

[B70] Murray R. J. Brosch T. Sander D. (2014). The functional profile of the human amygdala in affective processing: insights from intracranial recordings. *Cortex* 60 10–33. 10.1016/j.cortex.2014.06.010 25043736

[B71] Musso F. Konrad A. Vucurevic G. Schaffner C. Friedrich B. Frech P. (2006). Distributed BOLD-response in association cortex vector state space predicts reaction time during selective attention. *Neuroimage* 29 1311–1318. 10.1016/j.neuroimage.2005.07.059 16406256

[B72] Najstrom M. Jansson B. (2007). Skin conductance responses as predictor of emotional responses to stressful life events. *Behav. Res. Ther.* 45 2456–2463. 10.1016/j.brat.2007.03.001 17462590

[B73] Nishisato S. (1966). Reaction time as a function of arousal and anxiety. *Psychon. Sci.* 6 157–158. 10.3758/BF03328005

[B74] Northoff G. Richter A. Gessner M. Schlagenhauf F. Fell J. Baumgart F. (2000). Functional dissociation between medial and lateral prefrontal cortical spatiotemporal activation in negative and positive emotions: a combined fMRI/MEG study. *Cereb. Cortex* 10 93–107. 10.1093/cercor/10.1.93 10639399

[B75] Notebaert L. Crombez G. Van Damme S. De Houwer J. Theeuwes J. (2011). Signals of threat do not capture, but prioritize, attention: a conditioning approach. *Emotion* 11 81–89. 10.1037/a0021286 21401228

[B76] Ohman A. Flykt A. Esteves F. (2001). Emotion drives attention: detecting the snake in the grass. *J. Exp. Psychol. Gen.* 130 466–478. 10.1037/0096-3445.130.3.466 11561921

[B77] Oldfield R. C. (1971). The assessment and analysis of handedness: the Edinburgh inventory. *Neuropsychologia* 9 97–113. 10.1016/0028-3932(71)90067-4 5146491

[B78] Onnis R. Dadds M. R. Bryant R. A. (2011). Is there a mutual relationship between opposite attentional biases underlying anxiety? *Emotion* 11 582–594. 10.1037/a0022019 21668109

[B79] Oya H. Kawasaki H. Howard M. A. III Adolphs R. (2002). Electrophysiological responses in the human amygdala discriminate emotion categories of complex visual stimuli. *J. Neurosci.* 22 9502–9512. 12417674 10.1523/JNEUROSCI.22-21-09502.2002 PMC6758059

[B80] Paulus M. P. Stein M. B. (2006). An insular view of anxiety. *Biol. Psychiatry* 60 383–387. 10.1016/j.biopsych.2006.03.042 16780813

[B81] Penny W. D. Friston K. J. Ashburner J. T. Kiebel S. J. Nichols T. E. (2011). *Statistical Parametric Mapping: the Analysis of Functional Brain Images: The Analysis of Functional Brain Images*. New York, NY: Academic press.

[B82] Phelps E. A. Delgado M. R. Nearing K. I. LeDoux J. E. (2004). Extinction learning in humans: role of the amygdala and vmPFC. *Neuron* 43 897–905. 10.1016/j.neuron.2004.08.042 15363399

[B83] Phelps E. A. LeDoux J. E. (2005). Contributions of the amygdala to emotion processing: from animal models to human behavior. *Neuron* 48 175–187. 10.1016/j.neuron.2005.09.025 16242399

[B84] Phelps E. A. O’Connor K. J. Gatenby J. C. Gore J. C. Grillon C. Davis M. (2001). Activation of the left amygdala to a cognitive representation of fear. *Nat. Neurosci.* 4 437–441. 10.1038/86110 11276236

[B85] Pissiota A. Frans O. Michelgard A. Appel L. Langstrom B. Flaten M. A. (2003). Amygdala and anterior cingulate cortex activation during affective startle modulation: a PET study of fear. *Eur. J. Neurosci.* 18 1325–1331. 10.1046/j.1460-9568.2003.02855.x 12956731

[B86] Posse S. Wiese S. Gembris D. Mathiak K. Kessler C. Grosse-Ruyken M. L. (1999). Enhancement of BOLD-contrast sensitivity by single-shot multi-echo functional MR imaging. *Magn. Reson. Med.* 42 87–97. 10.1002/(SICI)1522-2594(199907)42:1<87::AID-MRM13>3.0.CO;2-O 10398954

[B87] Rabbitt P. Osman P. Moore B. Stollery B. (2001). There are stable individual differences in performance variability, both from moment to moment and from day to day. *Q. J. Exp. Psychol. A* 54 981–1003. 10.1080/02724980042000534 11765745

[B88] Reidy J. Richards A. (1997). A memory bias for threat in high-trait anxiety. *Pers. Individ. Dif.* 23 653–663. 10.1016/S0191-8869(97)00071-8

[B89] Rhudy J. L. Meagher M. W. (2000). Fear and anxiety: divergent effects on human pain thresholds. *Pain* 84 65–75. 10.1016/S0304-3959(99)00183-9 10601674

[B90] Rinck M. Reinecke A. Ellwart T. Heuer K. Becker E. S. (2005). Speeded detection and increased distraction in fear of spiders: evidence from eye movements. *J. Abnorm. Psychol.* 114 235–248. 10.1037/0021-843x.114.2.235 15869354

[B91] Salthouse T. A. Berish D. E. (2005). Correlates of within-person (across-occasion) variability in reaction time. *Neuropsychology* 19 77–87. 10.1037/0894-4105.19.1.77 15656765

[B92] Satpute A. B. Mumford J. A. Naliboff B. D. Poldrack R. A. (2012). Human anterior and posterior hippocampus respond distinctly to state and trait anxiety. *Emotion* 12 58–68. 10.1037/a0026517 22309734

[B93] Schmidt L. J. Belopolsky A. V. Theeuwes J. (2015). Attentional capture by signals of threat. *Cogn. Emot.* 29 687–694. 10.1080/02699931.2014.924484 24899117

[B94] Soares S. C. Esteves F. Flykt A. (2009). Fear, but not fear-relevance, modulates reaction times in visual search with animal distractors. *J. Anxiety Disord.* 23 136–144. 10.1016/j.janxdis.2008.05.002 18565724

[B95] Somerville L. H. Whalen P. J. Kelley W. M. (2010). Human bed nucleus of the stria terminalis indexes hypervigilant threat monitoring. *Biol. Psychiatry* 68 416–424. 10.1016/j.biopsych.2010.04.002 20497902 PMC2921460

[B96] Stelmack R. M. Houlihan M. Mcgarryroberts P. A. (1993). Personality, Reaction-Time, and Event-Related Potentials. *J. Pers. Soc. Psychol.* 65 399–409. 10.1037/0022-3514.65.2.399

[B97] Stujenske J. M. Likhtik E. Topiwala M. A. Gordon J. A. (2014). Fear and safety engage competing patterns of theta-gamma coupling in the basolateral amygdala. *Neuron* 83 919–933. 10.1016/j.neuron.2014.07.026 25144877 PMC4141236

[B98] Sullivan G. Apergis J. Bush D. Johnson L. R. Hou M. Ledoux J. (2004). Lesions in the bed nucleus of the stria terminalis disrupt corticosterone and freezing responses elicited by a contextual but not by a specific cue-conditioned fear stimulus. *Neuroscience* 128 7–14. 10.1016/j.neuroscience.2004.06.015 15450349

[B99] Van Den Berg J. Neely G. (2006). Performance on a simple reaction time task while sleep deprived. *Percept. Mot. Skills* 102 589–599. 10.2466/Pms.102.2.589-599 16826680

[B100] van Reekum C. M. Urry H. L. Johnstone T. Thurow M. E. Frye C. J. Jackson C. A. (2007). Individual differences in amygdala and ventromedial prefrontal cortex activity are associated with evaluation speed and psychological well-being. *J. Cogn. Neurosci.* 19 237–248. 10.1162/jocn.2007.19.2.237 17280513

[B101] Vlemincx E. Van Diest I. Van den Bergh O. (2015). Emotion, sighing, and respiratory variability. *Psychophysiology* 52 657–666. 10.1111/psyp.12396 25524012

[B102] Vuilleumier P. Armony J. L. Driver J. Dolan R. J. (2001). Effects of attention and emotion on face processing in the human brain: an event-related fMRI study. *Neuron* 30 829–841. 10.1016/S0896-6273(01)00328-2 11430815

[B103] Walker D. L. Toufexis D. J. Davis M. (2003). Role of the bed nucleus of the stria terminalis versus the amygdala in fear, stress, and anxiety. *Eur. J. Pharmacol.* 463 199–216. 10.1016/S0014-2999(03)01282-2 12600711

[B104] Weinstein A. M. (1995). Visual ERPs evidence for enhanced processing of threatening information in anxious university-students. *Biol. Psychiatry* 37 847–858. 10.1016/0006-3223(94)00249-3 7548459

[B105] Weiss A. D. (1965). The locus of reaction-time change with set, motivation, and age. *J. Gerontol.* 20 60–64. 14246532 10.1093/geronj/20.1.60

[B106] Welford A. T. (1980). “Choice reaction time: basic concepts,” in *Reaction Times*, ed. Welford A. T. (New York, NY: Academic Press), 73–128.

[B107] Whelan R. (2010). Effective analysis of reaction time data. *Psychol. Rec.* 58:9.

[B108] Whisman M. A. Richardson E. D. (2015). Normative data on the beck depression inventory–second edition (BDI-II) in college students. *J. Clin. Psychol.* 71 898–907. 10.1002/jclp.22188 25950150

[B109] Yiend J. Mathews A. (2001). Anxiety and attention to threatening pictures. *Q. J. Exp. Psychol. A* 54 665–681. 10.1080/02724980042000462 11548029

[B110] Zaitsev M. Hennig J. Speck O. (2004). Point spread function mapping with parallel imaging techniques and high acceleration factors: fast, robust, and flexible method for echo-planar imaging distortion correction. *Magn. Reson. Med.* 52 1156–1166. 10.1002/Mrm.20261 15508146

