# Supplementary material for: Amygdala fMRI Signal as a Predictor of Reaction Time
Source: Front Hum Neurosci. 2016 Oct 13;10:516. doi: 10.3389/fnhum.2016.00516 (PMC5061816; doi:10.3389/fnhum.2016.00516)
Supplement: Supplementary file 1 [file Data_Sheet_1.docx]

Supplementary Material

**Amygdala fMRI Signal as a Predictor of Reaction Time**

**Philipp Riedel, Mark J. Jacob, Dirk K. Müller, Nora C. Vetter, Michael N. Smolka^*^, Michael Marxen**

*** Correspondence:** Michael N. Smolka: [michael.smolka@tu-dresden.de](mailto:michael.smolka@tu-dresden.de)

# Supplementary Material and Methods

## Data analysis – Behavioral

The first RT trial of the RT-CT (always within the safe condition) was excluded as an outlier for all participants. Although the first safe trial was within three standard deviations (SD) of the individual RTs, the group mean showed to be much higher than the group mean of all other trials (> 3 SD). This can be explained by the observation that individuals all showed trials with slower responses across all the RT trials that were still within 3 SD. Which RT trials brought about slower responses varied across individuals, with the exception of the first trial that consistently elicited rather slow responses in all individuals. We assume that participants were not yet accommodated to the task on the first trial naturally resulting in slower reactions. This effect biased results in favor of hypothesis 1: main effect of condition [means: F(1,43) = 7.7, p = .008; medians: F(1,43) = 3.1, p = .089] and condition * subgroup interaction [means: F(1,43) = 11.2, p = .002; medians: F(1,43) = 7.0, p = .011]. To discard the observed effect as a potential confound, the first RT trial was generally excluded.

## Data analysis – Neuroimaging

### First-level analysis:

#### Exclusion of blocks or block onsets in Model 2 and Model 3

Because the first modelling approach (Model 1) did neither reveal a main effect of condition (threat/safe) for blocks nor for events on amygdala activity (SVC at uncorrected p < .05, KE ≥ 8 voxels) nor a main effect of condition on activity in any other brain region (whole-brain level at uncorrected p < .05, KE ≥ 8 voxels), we did not include regressors for threat-blocks, shock-blocks, safe-block onsets and threat-block onsets (as in Model 1) in Model 2 and Model 3. Another reason to not include these four conditions was our intent to analyze whether BOLD fluctuations independent of their cause correlate with the RTs. A reduction of variance by additional conditions in the GLM therefore proved counter-productive.

#### Model 3 [Correlation of the BOLD signal in the amygdala prior to a CRT trial with the trial-specific RT (Hypothesis 3)]:

To post-hoc confirm the results of the regressor model (Model 2) on a neuronal level, a HRF-convoluted model was applied (see Fig. S1A). The canonical HRF peaks at around 5 s after neuronal activation. Therefore we defined ‘virtual’ events at 2 TRs (i.e., ~5 s) before the onset of the regressor model, that is, at 3 TRs (i.e., ~7.5 s) prior to task onset. These neural events were parametrically modulated with the corresponding RT and convolved with the HRF in SPM. The model also included one separate nuisance regressor for the five shock events. We did not include blocks or block onsets in Model 3. However, to improve interpretation we performed an additional analysis with blocks and block onsets included in addition to the parametrically modulated ‘virtual’ event at -3TR. In that case, the BOLD-RT correlation could not be replicated. This finding is not surprising, because it is plausible that conditions did have some effect on amygdala activation that was not detected in the current study. However, it also highlights that the BOLD-RT correlation found in this study was driven by the experimental setup including a continuous threat. In an additional and subsequent approach to Model 3, separate GLMs with ‘virtual’ events from -5TR to +5TR (i.e., ~12.7 s prior to or after the CRT task) were computed (Fig. S2A) to illustrate the BOLD-RT correlation over time for the peak voxel (Fig. S1B).

# Supplementary Results

## Analysis without physiological noise correction

### Questionnaires:

The BDI score correlated with RTs independent of condition (means: r = .423, p = .004; medians: r = .469, p = .001). The high-anxious subgroup had a higher mean BDI score compared to the low-anxious subgroup [one-tailed independent samples t-test: t(43) = 2.211, p < .016]. However, the BDI score did not explain a difference of RTs between conditions, that is, repeated measure ANCOVA with the within-subject factor condition (threat/safe), the between-subject factor STAI-T subgroup (HTA/LTA) and the BDI score as covariate did not reveal a condition * BDI interaction [means: F(1,42) = .4, p = .512; medians: F(1,42) = 1.0, p = .324]. The condition * subgroup interaction reported for Hypothesis 1 was not affected when accounting for the BDI score [means: F(1,42) = 7.2, p = .010; medians: F(1,42) = 4.0, p = .051]. The PSS was also negatively correlated with the mean difference between RTs in the threat blocks as compared to the safe blocks (r = -.318, p = .033).

### Effect of condition on RTs (Hypothesis 1):

Analysis of the neuroimaging data for all participants (N = 45) was again performed with motion correction, but without incorporating additional eighteen regressors for cardiac and respiratory noise at subject level (Model 1). Analysis revealed no main effect of condition (threat/safe) on amygdala activity (SVC at uncorrected p < .01, KE ≥ 4).

### Correlation of the BOLD signal in the amygdala prior to a CRT trial with the trial-specific RT (Hypothesis 3):

Analysis of the neuroimaging data was again performed with motion correction, but without incorporating physiological noise correction at subject level (for Model 3). Analysis revealed a significant positive trial-by-trial correlation between BOLD activation and subsequent RTs irrespective of condition (threat/safe). The BOLD-RT correlation at a ‘virtual’ event 3 TRs (i.e., ~7.5 s) prior to the onset of the RT task was found for the same voxel within the amygdala (MNI: 30/-2/-22 after SVC) that was found for data analysis including physiological noise correction (see Results; Fig 4B). In contrast, p-values after SVC reflected more significant results when correction for physiological noise was not included: for the whole sample (FWE_corr_ p = .039) and for the 36 participants that had valid peripheral physiological measures for physiological noise correction (FWE_corr_ p = .002).

However, without physiological noise correction, whole brain analysis (performed for quality assurance) showed that peak voxels of the activation clusters (which also included the amygdala) were not located within the amygdala [according to the MNI to Talairach mapping atlas from the Yale BioImage Suite Package New Haven, Connecticut, USA; (see also Lacadie *et al.*, 2008)] for the whole group (N=45). For the subsample (N=36) the peak voxel was located within the amygdala both before and after physiological noise correction.

To conclude, we suggest that physiological noise correction reliably reduced cardiac and respiratory noise, but on the other hand also reduced statistical power of the observed effect.

There was no correlation between individual betas (i.e., correlation coefficients) retrieved after subject-level analysis (Model 3) for the peak group voxel and the individual STAI-T score (all p > .2).

## Analysis with physiological noise correction

### Effect of condition on amygdala BOLD signal (Hypothesis 2)

SPM ROI analysis revealed no significant main effect of condition (threat/safe) bilateral insula activity.

### Correlation of the BOLD signal in the amygdala prior to a CRT trial with the trial-specific RT (Hypothesis 3)

SPM ROI analysis revealed no significant positive trial-by-trial correlation between BOLD activation within the bilateral insula.

# Supplementary Discussion

In line with previous findings (Endler *et al.*, 1992; Hill *et al.*, 2013; Isobe *et al.*, 2014) we found a correlation of trait anxiety with self-ratings of depressive symptoms (BDI). Whether this correlation is caused by vulnerability factors common to anxiety and depression (reviewed in Andrews, 1996; Grupe and Nitschke, 2013) or by a predisposition to one condition by the other cannot be resolved within the current design. Critically, the current study supports the view that anxiety is a distinct condition that can be distinguished from depressive symptoms in healthy samples (Nitschke *et al.*, 2001), that is, our results show that trait anxiety (STAI-T) affects RTs in threat compared to safe conditions while depressive symptoms (BDI) do not. In addition, we found an increase of RTs irrespective of condition with the level of self-reported depressive symptoms (BDI). Although this result is in line with the consensus of cognitive impairment in depression, scientific findings on a correlation of impairment and depression severity are inconsistent (for a meta-analysis see McDermott and Ebmeier, 2009). Although RTs have been shown to be increased with depressive severity (Byrne, 1976; Austin *et al.*, 1999), other studies reported that BDI score only accounts for a small amount of variability in task performance (Delis *et al.*, 1994). Therefore a generalization of the BDI-RT correlation found in the current design on the population level cannot be undertaken.

An extended network including, for example, the amygdala, the BNST, the hippocampus, the vmPFC, the insula and the primary motor cortex, has been identified for the processing of sustained threat. Whole brain analysis in the current study did not show activation in further brain regions associated with sustained threat processing. ROI-analysis for hippocampus and vmPFC seemed not appropriate. Previous involvement of hippocampal activation has been predominantly shown during the acquisition phase of human contextual threat conditioning (Marschner *et al.*, 2008; Andreatta *et al.*, 2015) with rather few studies showing an involvement during return of threat as in the current study (Lonsdorf *et al.*, 2014). Also the hippocampus has been discussed to rather track and guide one’s location in complex environments that are clearly to differentiate (i.e., virtual reality) (Fanselow and Dong, 2010; Andreatta *et al.*, 2015). Even though activation was also found for slight changes in these contexts (Baeuchl *et al.*, 2015), it is unlikely that the stimuli in this study recruited the hippocampus. vmPFC activation has been particularly shown for threat extinction (Kalisch *et al.*, 2006), which does not apply to our design (also see Discussion).

Preprocessing included slice time correction to the middle slice. Given a TR of 2.54 s, the effective time of acquisition for each block would be 1.27 s later. Therefore some effect of trial response cannot be excluded. However, if trial response would be the cause that drives the correlation than we should find an even stronger effect when sampling 1 TR later. But this was not the case. Therefore we are positive that the correlation results from pre-trial amygdala activity changes.

# Supplementary Figures and Tables

## Supplementary Figures


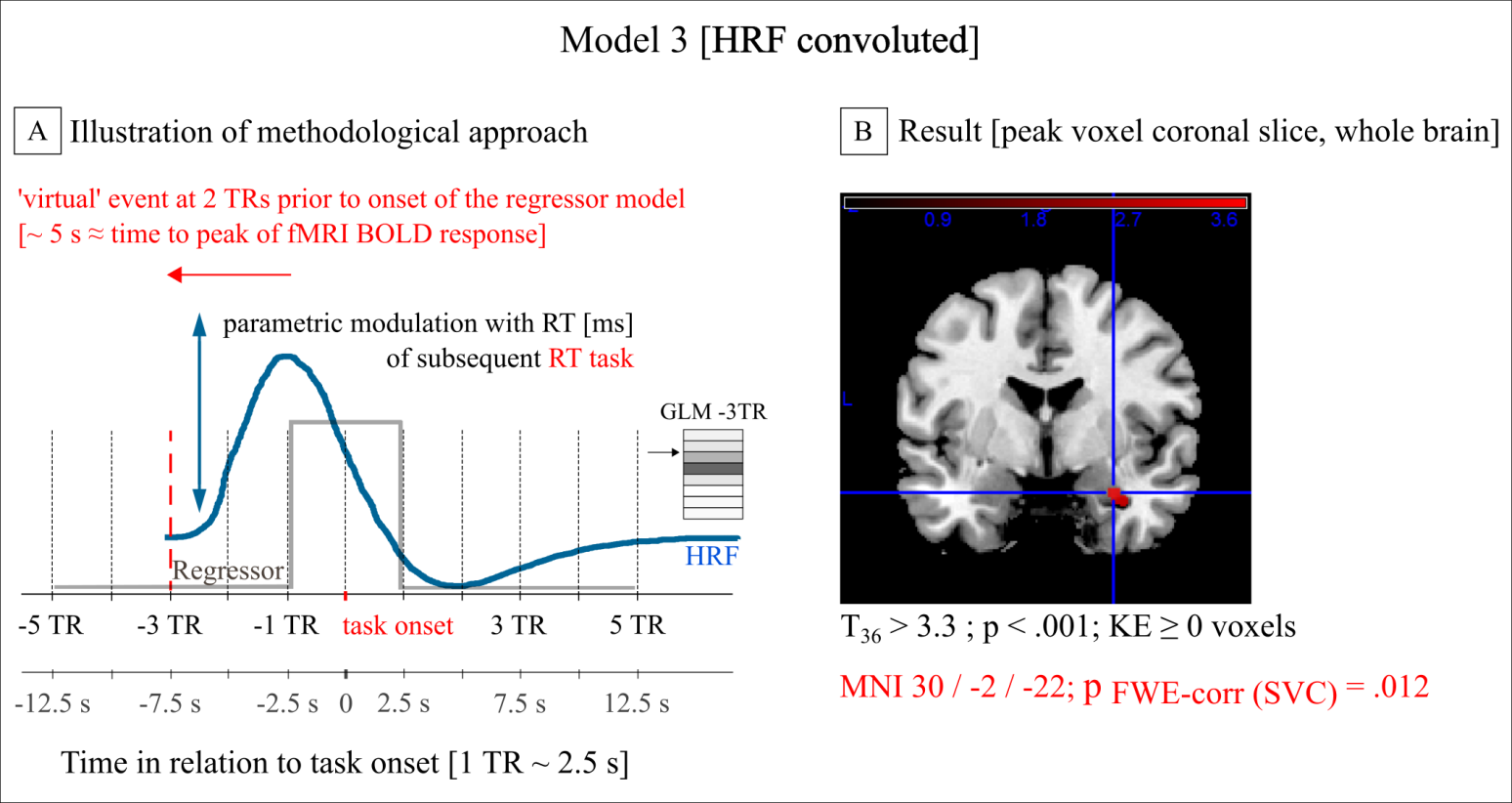


**Supplementary Figure 1:** Supplementary neuroimaging data analysis. [A] Graphical illustration of the first-level neuronal model. The HRF-convoluted model was used to post-hoc confirm the results of the regressor model. Because the canonical HRF (and therefore the BOLD-signal) peaks at around 5 s after neuronal activation, we defined a ‘virtual’ event at 2 TRs (i.e., ~5 s) before the onset of the regressor model, that is, at 3 TRs (i.e., ~7.5 s) prior to task onset. ‘Virtual’ events were parametrically modulated by the trial-specific RT. [B] Results of the second-level analysis. Amygdala signal at about 7.5 s prior to the CRT task correlated with the RT of the corresponding trial. The anatomical illustration shows a coronal slice overlaid with the t-map at whole brain level. The result of the statistical analysis is presented after small volume correction (SVC).


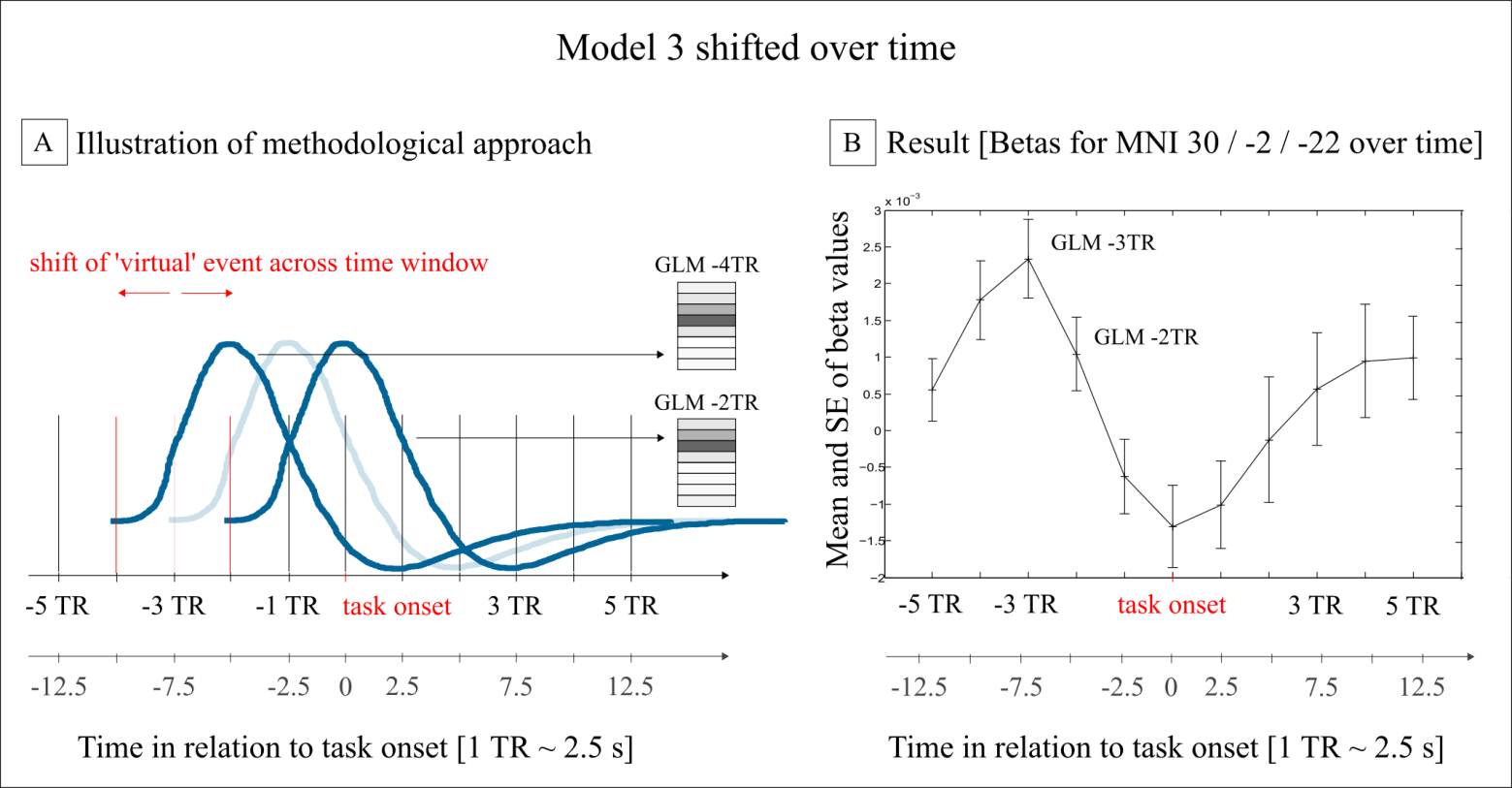


**Supplementary Figure 2:** [A1] Separate first-level HRF-convoluted models (Model 3) over time with different offsets from -5TR to +5TR (i.e., ~12.7 s prior to or after the CRT task) were computed. [B] Mean beta values for the peak voxel found at -3TR (MNI: 30/-2/-22) were extracted and graphically illustrated over time with standard errors.

## Supplementary Tables

**Supplementary Table 1: Mean scores (M) and standard deviations (SD) for self-reported questionnaires in the whole sample (N = 45).**

|  |  |  |
| --- | --- | --- |
| **Questionnaire** | **M** | **SD** |
| BDI | 4.51 | 4.19 |
| PSS | 12.40 | 5.05 |
| STAI_State_ | 36.13 | 5.98 |
| STAI_Trait_ | 36.24 | 6.14 |

# References

Andreatta, M., Glotzbach-Schoon, E., Muhlberger, A., Schulz, S.M., Wiemer, J., and Pauli, P. (2015). Initial and sustained brain responses to contextual conditioned anxiety in humans. *Cortex* 63**,** 352-363. doi: 10.1016/j.cortex.2014.09.014.

Andrews, G. (1996). Comorbidity in neurotic disorders: The similarities are more important than the differences. *Current controversies in the anxiety disorders***,** 3-20.

Austin, M.P., Mitchell, P., Wilhelm, K., Parker, G., Hickie, I., Brodaty, H. et al. (1999). Cognitive function in depression: a distinct pattern of frontal impairment in melancholia? *Psychol. Med.* 29**,** 73-85.

Baeuchl, C., Meyer, P., Hoppstadter, M., Diener, C., and Flor, H. (2015). Contextual fear conditioning in humans using feature-identical contexts. *Neurobiol. Learn. Mem.* 121**,** 1-11. doi: 10.1016/j.nlm.2015.03.001.

Byrne, D.G. (1976). Choice reaction times in depressive states. *Br. J. Soc. Clin. Psychol.* 15**,** 149-156.

Delis, D.C., Kramer, J.H., Kaplan, E., and Ober, B.A. (1994). *CVLT-C: California Verbal Learning Test.*

Endler, N.S., Cox, B.J., Parker, J.D., and Bagby, R.M. (1992). Self-reports of depression and state-trait anxiety: Evidence for differential assessment. *J. Pers. Soc. Psychol.* 63**,** 832.

Fanselow, M.S., and Dong, H.W. (2010). Are the dorsal and ventral hippocampus functionally distinct structures? *Neuron* 65**,** 7-19. doi: 10.1016/j.neuron.2009.11.031.

Grupe, D.W., and Nitschke, J.B. (2013). Uncertainty and anticipation in anxiety: an integrated neurobiological and psychological perspective. *Nat. Rev. Neurosci.* 14**,** 488-501. doi: 10.1038/nrn3524.

Hill, B.D., Musso, M., Jones, G.N., Pella, R.D., and Gouvier, W.D. (2013). A Psychometric Evaluation of the STAI-Y, BDI-II, and PAI Using Single and Multifactorial Models in Young Adults Seeking Psychoeducational Evaluation. *J. Psychoeduc. Assess.* 31**,** 300-312.

Isobe, K., Ishizu, T., Oikawa, H., Kamimaki, T., Ishijima, M., Nanmoku, T. et al. (2014). Correlation between blood biomarkers and depression and anxiety scales in apparently healthy individuals. *Int J Anal Bio-Sci Vol* 2.

Kalisch, R., Korenfeld, E., Stephan, K.E., Weiskopf, N., Seymour, B., and Dolan, R.J. (2006). Context-dependent human extinction memory is mediated by a ventromedial prefrontal and hippocampal network. *J. Neurosci.* 26**,** 9503-9511. doi: 10.1523/Jneurosci.2021-06.2006.

Lacadie, C., Fulbright, R., Arora, J., Constable, R., and Papademetris, X. (Year). "Brodmann Areas defined in MNI space using a new Tracing Tool in BioImage Suite", in: *Proceedings of the 14th Annual Meeting of the Organization for Human Brain Mapping*), 771.

Lonsdorf, T.B., Haaker, J., and Kalisch, R. (2014). Long-term expression of human contextual fear and extinction memories involves amygdala, hippocampus and ventromedial prefrontal cortex: a reinstatement study in two independent samples. *Soc. Cogn. Affect. Neurosci.* 9**,** 1973-1983. doi: 10.1093/scan/nsu018.

Marschner, A., Kalisch, R., Vervliet, B., Vansteenwegen, D., and Buchel, C. (2008). Dissociable roles for the hippocampus and the amygdala in human cued versus context fear conditioning. *J. Neurosci.* 28**,** 9030-9036. doi: 10.1523/Jneurosci.1651-08.2008.

McDermott, L.M., and Ebmeier, K.P. (2009). A meta-analysis of depression severity and cognitive function. *J. Affect. Disord.* 119**,** 1-8. doi: 10.1016/j.jad.2009.04.022.

Nitschke, J.B., Heller, W., Imig, J.C., McDonald, R.P., and Miller, G.A. (2001). Distinguishing dimensions of anxiety and depression. *Cognit. Ther. Res.* 25**,** 1-22.
